# Pulmonary Sequelae of Severe Acute COVID‐19 and Multisystem Inflammatory Syndrome (MIS‐C) in Dutch Children

**DOI:** 10.1002/ppul.71426

**Published:** 2025-12-15

**Authors:** Lieke C. E. Noij, Caroline L. H. Brackel, Mariëlle W. Pijnenburg, Michiel A. G. E. Bannier, Sanne F. Kloosterman, Joost G. van den Aardweg, Arjen Pelgröm, Irene Kuipers, Erik G. J. von Asmuth, Emilie P. Buddingh, Miriam G. Mooij, Angelique M. A. M. Winkel, Lotte Haverman, Lorynn Teela, Anke H. Maitland van der Zee, Johannes B. van Goudoever, Simone Hashimoto, Suzanne W. J. Terheggen‐Lagro

**Affiliations:** ^1^ Department of Pediatrics, Division of Pediatric Pulmonology and Allergy, Emma Children's Hospital Amsterdam University Medical Center, University of Amsterdam Amsterdam the Netherlands; ^2^ Department of Pediatrics Tergooi Medical Centrum Hilversum the Netherlands; ^3^ Department of Pediatrics, Division of Pediatric Respiratory Medicine and Allergology, Sophia Children's Hospital Erasmus Medical Center Rotterdam the Netherlands; ^4^ Department of Pediatrics, Division of Pediatric Respiratory Medicine, MosaKids Children's Hospital Maastricht University Medical Center Maastricht the Netherlands; ^5^ Department of Pulmonary Medicine Amsterdam University Medical Center, University of Amsterdam Amsterdam the Netherlands; ^6^ Department of Pediatrics, Division of Pediatric Cardiology, Emma Children's Hospital Amsterdam University Medical Center, University of Amsterdam Amsterdam the Netherlands; ^7^ Department of Pediatrics, Willem‐Alexander Children's Hospital Leiden University Medical Center Leiden the Netherlands; ^8^ Department of Pediatrics, Division of Pediatric Nephrology, Sophia Children's Hospital Erasmus Medical Center, Rotterdam the Netherlands; ^9^ Department of Pediatrics Spaarne Gasthuis Hoofddorp and Haarlem the Netherlands; ^10^ Department of Child and Adolescent Psychiatry & Psychosocial Care, Emma Children's Hospital Amsterdam University Medical Center, University of Amsterdam Amsterdam the Netherlands; ^11^ Department of Pediatrics, Emma Children's Hospital Amsterdam University Medical Center, University of Amsterdam Amsterdam the Netherlands

**Keywords:** acute COVID‐19, children, cardiopulmonary exercise test (CPET), MIS‐C, Pulmonary sequelae

## Abstract

**Background:**

Although rare, COVID‐19 in children may lead to hospitalization due to severe respiratory symptoms, or a hyperinflammatory state called Multisystem Inflammatory Syndrome in Children (MIS‐C). This study examined respiratory morbidity in children 5 to 12 months after hospitalization for MIS‐C or COVID‐19.

**Methods:**

In this multi‐center, prospective cohort study, children (0–17 years) with a history of hospitalization for MIS‐C or COVID‐19 in Dutch hospitals were invited for follow‐up. Visits were scheduled in one of three academic hospitals, to evaluate current clinical status and health‐related quality of life (HRQoL), and perform lung function tests and cardiopulmonary exercise testing (CPET).

**Results:**

72 children were included (43 MIS‐C, 29 COVID‐19), of whom 19% (5% and 41%, respectively) reported long‐term respiratory symptoms including dyspnea and cough, a median of 8 months after hospitalization. Fatigue was the most common non‐respiratory symptom. HRQoL was more frequently (60%) impaired in the COVID‐19 group than the MIS‐C group (14%). Spirometry (*n* = 48) and CPET (*n* = 40) were conducted in children aged > 4 and > 6 years, respectively. Spirometry was abnormal in 15% of the MIS‐C group and 44% of COVID‐19 group, CPET in 41% and 75%, respectively. Deconditioning patterns were the most common reason (30%) for abnormal CPET results.

**Conclusion:**

Long‐term respiratory sequelae and fatigue occurred after both MIS‐C and severe COVID‐19, but respiratory symptoms and impaired HRQoL were more frequent after COVID‐19. Lung function and CPET abnormalities in children with COVID‐19 often corresponded with symptoms. Children with MIS‐C often showed CPET abnormalities without respiratory complaints or lung function changes.

## 1 Introduction

Severity of COVID‐19 in children is dependent on age, comorbidities, and SARS‐CoV‐2 variant. Only 0.3%−0.9% of cases need hospital admission, with a higher admission rate in infants [1, 2, 3, 4, 5]. COVID‐19 presentation in children often consists of a combination of fever and upper respiratory symptoms such as cough, rhinitis and anosmia, while respiratory presentations such as shortness of breath, bronchiolitis, and acute respiratory distress syndrome (ARDS) or pneumonitis are rare [6, 7]. Additional symptoms that children can present with are fatigue, vomiting, diarrhea, and headache. Besides severe respiratory symptoms, SARS‐CoV‐2 infection may also lead to hospitalization due to Multisystem Inflammatory Syndrome in Children (MIS‐C), an often severe condition, which presents 4 to 6 weeks after acute infection. It is caused by a hyperinflammatory, autoimmune response to SARS‐CoV‐2, and may be complicated by heart failure and ARDS [8, 9].

Respiratory pathogens such as respiratory syncytial virus (RSV) can lead to long‐term pulmonary morbidity and altered lung function trajectories [10]. This most likely also applies to SARS‐CoV‐2, with recent studies reporting long‐term sequelae in about one‐third of hospitalized children with COVID‐19 or MIS‐C [11]. Besides direct organ damage during the acute phase of both COVID‐19 and MIS‐C, long‐term sequelae may also result from the development of the post‐acute infectious syndrome called Pediatric Post‐COVID‐19 Condition (PPCC). PPCC is a chronic condition, with a wide range of persisting or new‐onset symptoms lasting at least 2 months, occurring within 3 months of acute SARS‐CoV‐2 infection, and which may fluctuate or relapse [12]. Possible causative factors of PPCC are viral persistence, endothelial dysfunction, coagulopathy, autonomic dysfunction, chronic inflammation, and autoimmunity [13]. Children suffering from more severe pediatric COVID‐19 have a higher chance of developing PPCC [9, 11]. Severity and duration of PPCC are heterogeneous, with most patients recovering after a few months, but some children experiencing symptoms up to 3 years after infection [14]. Common long‐term respiratory symptoms include cough, chest tightness, shortness of breath, and worsening of underlying asthma [15, 16]. Development of PPCC after MIS‐C has also been reported, although it is not yet clear whether the pathophysiology of PPCC is different after severe pediatric COVID‐19 or MIS‐C.

Until now, studies reporting on the prevalence of long‐term sequelae after severe COVID‐19 have mostly been based on patient‐reported symptoms. Only a few studies have focused on the assessment of long‐term morbidity in children admitted with either pediatric COVID‐19 or MIS‐C using pulmonary function testing and cardiopulmonary exercise testing (CPET) [17, 18, 19]. This study aimed to investigate long‐term respiratory morbidity in children who suffered from severe pediatric COVID‐19 or from MIS‐C, focusing on respiratory complaints, lung function testing, and CPET.

## 2 Materials and Methods

### 2.1 Study Design, Setting and Participants

The “Clinical features of COVID‐19 in Pediatric Patients, follow‐up” (COPP2) study was a multi‐center, prospective, observational cohort study, investigating long‐term pulmonary sequelae of pediatric COVID‐19 and MIS‐C. Children, aged 0 to 17 years old, previously enrolled in the “Clinical features of COVID‐19 in Pediatric Patients” (COPP) study [20] between March 2020 and April 2022, were eligible to participate. In short, the COPP‐study included children with COVID‐19 or MIS‐C (as defined by the World Health Organization [21]), who presented in one of 41 Dutch hospitals. For further information about inclusion criteria and study procedures of the COPP‐study, we refer to Tulling et al. [3]. For the COPP2‐study, children and/or caregivers who had provided consent to be approached for follow‐up studies were invited through email to participate. Those who responded to the email, were then approached by a researcher from the COPP2‐study team. Children included in the COPP2‐study underwent a study visit in one of three academic pediatric hospitals (Amsterdam UMC, Erasmus MC, and Maastricht UMC), 5 to 12 months after hospitalization. Only children admitted due to MIS‐C, or with severe COVID‐19 defined as respiratory symptoms requiring hospitalization, either for observation or medical support (e.g. medication or oxygen therapy), were included in this analysis. The study visit was postponed in case of active acute respiratory infection or symptoms, or exacerbation of underlying diseases. The COPP2‐study was evaluated and approved by the local medical regional ethics committee (METC Amsterdam, reference number N20.043 NL7473696.018.20). All participants ≥ 12 years and/or caregivers provided written informed consent.

### 2.2 Study Procedures

#### 2.2.1 Clinical Data Collection

Data on course and severity of acute COVID‐19 or MIS‐C (symptoms, hospitalization, and respiratory support), and clinical characteristics (medical history and comorbidities), were extracted from data collected in the COPP study. During the study visit, further demographic factors (age, sex) and clinical characteristics (current symptoms, physical examination) were collected (see Supporting Information – Appendix 1 for detailed description of when symptoms were considered long‐term). The suspected SARS‐CoV‐2 variant was determined by the dominant virus type in the Netherlands at the time of infection based on numbers from the National Institute for Public Health and the Environment (RIVM) [22]. The COPP2‐study used Castor Electronic Data Capture (www.castoredc.com) [23] and KLIK (www.hetklikt.nu) [24] for collection of data, questionnaires, and storage. Both are external parties with whom the Amsterdam UMC has license agreements. This study was first designed to assess persistent respiratory complaints and fatigue.

#### 2.2.2 Quality of Life Questionnaires

Health‐related quality of life (HRQoL) was assessed by the Dutch Pediatric Quality of Life Inventory 4.0 (PedsQL 4.0) (self‐reported in children aged 8–18 years old). Higher scores indicate better functioning. Cut‐off points for at‐risk status of impaired HRQoL by the PedsQL 4.0 were set at 1 standard deviation (SD) below the Dutch pediatric population mean from 2021 [25], as described by Varni et al. [26]. More details concerning PedsQL questionnaires are published in Supporting Information – Appendix 1.

#### 2.2.3 Lung Function Tests

Spirometry with reversibility testing was performed in children older than 4 years at the time of study visit, according to the ATS/ERS guidelines of 2022 [27] and GLI reference values [28, 29]. Spirometry and body plethysmography results were reviewed by three independent pediatric pulmonologists to determine whether results were of sufficient quality to be included in this study. Results were categorized into normal and abnormal based on predefined limits (see Supporting Information – Appendix 1).

#### 2.2.4 Cardiopulmonary Exercise Testing (CPET)

CPET was performed in children older than 6 years at the time of the study visit. A detailed description of CPET protocol can be found in Supporting Information – Appendix 1. CPET results were scored on maximum effort, overall abnormalities, and abnormalities for specific domains. Variables included per domain were:

− Cardiovascular responses: peak oxygen pulse (abnormal: O_2_ pulse < 80%), difference in systolic pressure (Psys), and ratio of delta oxygen uptake to delta work rate (abnormal: ΔV'O2/ΔWR < 8.3).
− Respiratory responses: ratio of tidal volume at maximum exercise to vital capacity (abnormal: VT/VC > 65%) and ratio of inspiratory time to total respiratory cycle time (abnormal: Ti/Tot < 45%).
− Breathing regulation (controlled by the central nervous system): peak breath frequency (abnormal: BF z‐score ≥ 1.96), BF‐slope, and PETCO_2_ ‐slope.
− Deconditioning pattern (a complex process of physiological change, usually following a period of inactivity): abnormal oxygen uptake at anaerobic threshold (abnormal: V'O2‐AT < 40%).
− Gas exchange in the lungs: respiratory exchange ratio (abnormal: RER > 1.14), difference in oxygen saturation (SpO2), ventilation minute for carbon dioxide production at anaerobic threshold (abnormal: VE/VCO2 at AT z‐score ≥ 1.96), end‐tidal partial pressure for carbon dioxide (abnormal: PETCO2 z‐score ≥ 1.96), and Ratio of mixed‐expired and end‐tidal CO2 (abnormal: P(ET‐E)CO2/PETCO2 > 35%).

Other cut‐off values can be found in Supporting Information – Appendix 1.

### 2.3 Statistical Analyses

Statistical analyses were performed for the total group of participants, as well as subgroups of participants based on acute disease presentation (MIS‐C or COVID‐19). The primary outcome was the percentage of children with self‐reported respiratory symptoms 5 to 12‐months following MIS‐C or COVID‐19. Secondary outcomes were (type of) abnormalities in physical examination, HRQoL, lung function, and CPET.

IBM Statistical Package for Social Sciences (SPSS) version 28.0 and R (version 4.1.2) with RStudio (version 2021.09.1 + 372) were used to perform statistical analyses. Descriptive analyses were performed to summarize demographical and clinical characteristics and lung function and CPET results, displayed as mean and standard deviation (SD) for normally distributed variables, and median and interquartile range (IQR) for abnormally distributed variables, or when abnormal distribution was assumed. A one‐way analysis of covariance (ANCOVA) was conducted to compare the means of continuous variables between the two groups, and logistic regression analyses were conducted to compare binary variables, where the presence of previous pulmonary comorbidities was considered as covariate.

## 3 Results

### 3.1 Demographics

**Figure 1:**
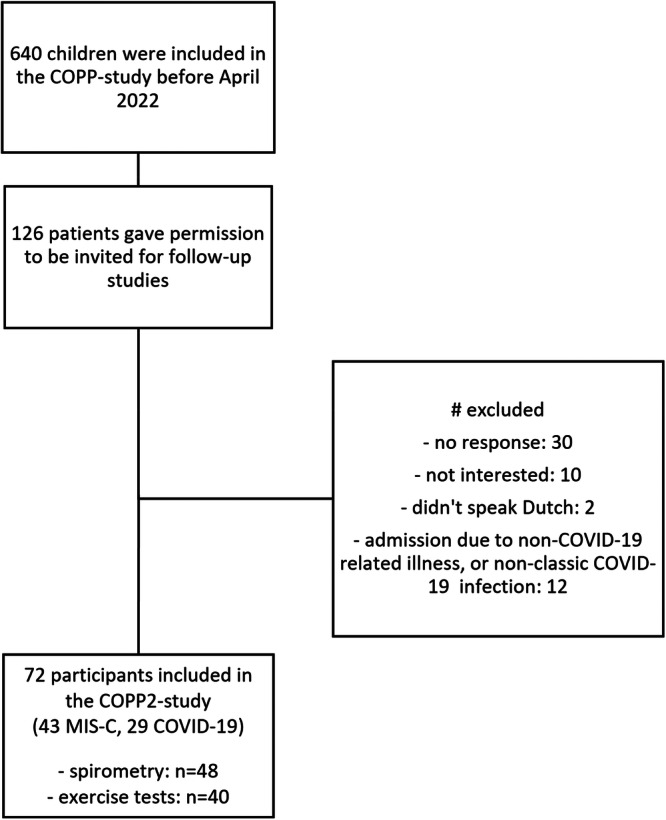
Inclusion procedure for the study cohort. COPP:, COVID in Pediatric Patients study. MIS‐C: Multisystem Inflammatory Syndrome in Children. Spirometry was performed in children > 4 years, CPET in children > 6 years.

Out of 126 children who participated in the COPP‐study and initially gave permission to be invited for follow‐up, 54 were excluded for various reasons, of which ‘no response’ was most common (*n* = 30) (Figure 1). Of the 72 participants included in this study, 43 children presented with MIS‐C and 29 children with COVID‐19. The demographical and clinical characteristics of these patients can be found in Table 1. Participants had a median age of 10 years (IQR: 3–10). The total cohort consisted of slightly more boys than girls (57% and 43%, respectively). Median time between hospital admission and study visit was 8 months (IQR: 6–10).

**Table 1:** Demographics and patient characteristics at time of follow‐up visit, grouped by acute COVID‐19 presentation.

	Total	MIS‐C	COVID‐19
	*n* = 72	*n* = 43	*n*= 29
**Age** (median, IQR)	10 (3, 13)	11 (8, 13)	2 (0, 13)
**Sex**			
Boy	41 (57%)	25 (58%)	16 (55%)
Girl	31 (43%)	18 (42%)	13 (45%)
**BMI** z‐score (median, IQR)	0.30 (−0.60, 1.31)	0.52 (−0.55, 1.47)	−0.18 (−0.78, 1.05)
**SARS‐CoV‐2 variant**			
Wild‐type	53 (74%)	30 (70%)	23 (79%)
Alpha	8 (11%)	5 (12%)	3 (11%)
Delta	10 (14%)	8 (19%)	2 (7%)
Omicron	1 (1%)	0 (0%)	1 (3%)
**Time between acute presentation and study visit** (in months, median + IQR)	8 (6, 10)	8 (7, 11)	7 (6, 9)
**Comorbidities**			
None	42 (58%)	31 (72%)	11 (38%)
Viral wheezing	4 (6%)	0 (0)	4 (14%)
Asthma	7 (10%)	3 (7%)	4 (14%)
Atopic syndrome	3 (4%)	2 (5%)	1 (3%)
Prematurity	3 (4%)	0 (0)	3 (10%)
Other pulmonary diseases^a^	6 (8%)	1 (2%)	5 (17%)
Other^a^	16 (22%)	6 (14%)	9 (31%)
**Most common symptoms in the acute phase** **(present in > 25% of children)**			
Fever	63 (88%)	43 (100%)	20 (69%)
Fatigue	38 (53%)	25 (58%)	13 (45%)
Nausea/vomiting	34 (47%)	30 (70%)	4 (14%)
Abdominal pain	34 (47%)	30 (70%)	4 (14%)
Headache	28 (39%)	23 (54%)	5 (17%)
Coughing	27 (38%)	3 (7%)	24 (83%)
Dyspnea	27 (38%)	6 (14%)	21 (72%)
Skin lesions	27 (38%)	26 (61%)	1 (3%)
Diarrhea	27 (38%)	23 (54%)	4 (14%)
**Hospital admission during the acute phase (yes)**	60 (83%)	42 (98%)	18 (62%)
Length of admission, in days (median, IQR)	7 (5, 10)	8 (6, 10)	4.5 (3, 7.5)
ICU admission (yes)	25 (35%)	23 (54%)	2 (7%)
**Respiratory support**			
Any	33 (46%)	21 (49%)	12 (41%)
Invasive mechanical ventilation	3 (4%)	3 (7%)	0 (0%)

*Note:* Data are presented as *n* (%), unless indicated otherwise.

a Other pulmonary diseases include: cystic fibrosis, tracheomalacia, etc. Other comorbidities include epilepsy, congenital CMV, IgA deficiency, myasthenia gravis, etc.

Abbreviations: BMI, Body Mass Index; ICU, Intensive Care Unit; IQR, Interquartile range; MIS‐C, Multisystem Inflammatory Syndrome in Children; SD, Standard Deviation.

For the MIS‐C group, the median age was 11 (IQR: 3–13) years. Most children had no comorbidities (72%). Twenty‐three children (54%) were admitted to the ICU in the acute phase.

For the COVID‐19 group, the median age was 2 (IQR: 0–13) years. Most children had comorbidities (62%), of which pulmonary comorbidities were most frequent (13/29, 45%). Two children (7%) required ICU admission during the acute phase.

### 3.2 Clinical Long‐Term Sequelae

Table 2 shows differences in clinical long‐term sequelae between the two groups, with statistical analyses adjusted for pulmonary comorbidities. In total, 14 children reported long‐term respiratory symptoms (5% MIS‐C group; 41% COVID‐19 group), of which dyspnea during exercise was the most prevalent (5% and 28%, respectively). Fatigue was the most commonly reported overall long‐term symptom (42% MIS‐C; 24% COVID‐19). Twelve (17%) children had one or more abnormalities during physical examination, of which tachypnea was most frequently objectified (12% MIS‐C; 7% COVID‐19).

**Table 2:** Long‐term clinical respiratory outcomes in children with either MIS‐C or COVID‐19.

	Total	MIS‐C	COVID‐19	*p*‐value^a^
	*n* = 72	*n* = 43	*n* = 29	
**Long‐term complaints: Respiratory symptoms**	14 (19%)	2 (5%)	12 (41%)	< 0.01
Dyspnea during exercise	10 (14%)	2 (5%)	8 (28%)	0.01
Dyspnea while resting	3 (4%)	1 (2%)	2 (7%)	0.40
Cough	6 (8%)	0 (0%)	6 (21%)	NA
Wheezing	1 (1%)	0 (0%)	1 (3%)	NA
**Long‐term complaints: Fatigue** b	25 (35%)	18 (42%)	7 (24%)	0.09
**Abnormalities during physical examination**				
Tachypnea	8 (10%)	5 (12%)	2 (7%)	0.79
Oxygen saturation < 94%	0 (0%)	0 (0%)	0 (0%)	N.A
Wheeze	3 (4%)	0 (0%)	3 (10%)	N.A
Rhonchi	5 (6%)	0 (0%)	3 (10%)	0.51
**Health related quality of life** **(PedsQL‐score)**	*n* = 45	*n* = 35	*n* = 10	
Total score (mean, SD)	79.11 (16.67)	83.85 (11.12)	61.52 (18.33)	< 0.01
*Impaired*	11 (24%)	5 (14%)	6 (60%)	< 0.01
Physical functioning	80.16 (20.14)	85.71 (13.09)	58.75 (24.60)	< 0.01
*Impaired*	17 (38%)	9 (26%)	8 (80%)	0.01
Emotional functioning	80.43 (17.73)	83.71 (15.14)	68.00 (20.30)	0.02
*Impaired*	9 (20%)	4 (11%)	5 (50%)	0.01
Social functioning	83.80 (16.17)	88.00 (12.76)	68.50 (14.54)	< 0.01
*Impaired*	8 (18%)	3 (9%)	5 (50%)	< 0.01
School functioning	71.41 (20.59)	76.86 (15.75)	52.50 (19.76)	< 0.01
*Impaired*	16 (36%)	9 (26%)	7 (70%)	0.03
Psychosocial functioning	78.55 (16.00)	82.86 (11.56)	63.00 (16.38)	< 0.01
*Impaired*	9 (20%)	4 (11%)	5 (50%)	0.01

*Note:* Data are presented as *n* (%), unless indicated otherwise.

a *p*‐value for dichotomous variables from logistic regression (with covariate pulmonary comorbidities), *p*‐value for continuous variables from ANCOVA (with covariate pulmonary comorbidities).

^b^ Other non‐respiratory symptoms are described in supporting table S1.

Abbreviations: MIS‐C, multisystem Inflammatory Syndrome in Children; NA, not applicable; PedsQL, Pediatric Quality of Life Inventory; SD, standard deviation.

Sub‐analyses for two age groups (0‐5 years and 6‐18 years) are presented in Table S1. Younger children most often presented with COVID‐19 (81%), and older children mostly with MIS‐C (77%). There was a trend towards more long‐term respiratory symptoms in younger children compared to older children (33% *vs.* 14%), although results were not statistically significant.

Table S2 displays additional information on non‐respiratory long‐term symptoms for 51 children.

### 3.3 Health‐Related Quality of Life

Forty‐five children aged ≥ 8 years completed the PedsQL 4.0 questionnaire (Table 2). Children in the COVID‐19 group scored significantly worse on all domains of HRQoL. Sixty percent of children from this group reported impaired HRQoL, compared to 14% from the MIS‐C group (*p* < 0.01).

### 3.4 Lung Function Tests

Five percent of the MIS‐C group and 66% of the COVID‐19 group were too young to perform lung function testing. In total, 50 children performed spirometry, of which 48 were deemed of technically sufficient quality, and 39 (54%) performed plethysmography (Table 3). Due to low power, no statistical tests were performed to compare the two groups.

**Table 3:** Spirometry, body plethysmography, and CPET at study visit.

	Total		MIS‐C		COVID‐19	
Spirometry prebronchodilator	*n* = 48		*n* = 39		*n* = 9	
FEV_1_						
% Predicted (mean, SD)^a^	95%	(±11)	96%	(±11)	89%	(±11)
Z‐score (median, IQR)^a^	−0.34	(−1.08, 0.30)	−0.30	(−0.95, 0.40)	−1.03	(−1.88, −0.31)
Abnormal (*n*, %)	6	(13%)	3	(8%)	3	(33%)
FVC						
% Predicted	99%	(±12)	99%	(±12)	96%	(±12)
Z‐score	−0.08	(−0.74, 0.45)	−0.04	(−0.71, 0.46)	−0.42	(−1.19, 0.14)
abnormal (*n*, %)	2	(4%)	2	(5%)	0	(0%)
FEV_1_/FVC						
Absolute value	0.84	(±0.05)	0.85	(±0.05)	0.81	(±0.06)
% Predicted	96%	(±7)	96%	(±7)	94%	(±7)
Z‐score	−0.59	(−1.14, −0.06)	−0.55	(−1.00, 0.14)	−1.15	(−1.61, −0.37)
Abnormal (*n*, %)	4	(8%)	2	(5%)	2	(22%)
FEF25_75						
% Predicted	83%	(±18)	86%	(±17)	71%	(±15)
Z‐score	−0.78	(−1.33, −0.25)	−0.70	(−1.21, −0.20)	−1.50	(−1.93, −0.59)
Abnormal (*n*, %)	8	(17%)	4	(10%)	4	(44%)
**Spirometry postbronchodilator**	*n* = 47		*n* = 38		*n* = 9	
FEV_1_						
% Predicted	100%	(±11)	101%	(±11)	96%	(±12)
Z‐score	0.09	(−0.59, 0.60)	0.10	(−0.56, 0.65)	−0.27	(−1.32, 0.40)
Abnormal (*n*, %)	3	(6%)	3	(8%)	0	(0%)
Reversibility ≥ 10% (*n*, %)	4	(8%)	2	(5%)	2	(22%)
FVC						
% Predicted	101%	(±12)	100%	(±12)	101%	(±13)
Z‐score	0.06	(−0.71, 0.65)	0.06	(−0.68, 0.65)	0.06	(−0.78, 0.25)
Abnormal (*n*, %)	2	(4%)	2	(5%)	0	(0%)
FEV_1_/FVC						
Absolute value	0.88	(±0.05)	0.88	(±0.05)	0.84	(±0.05)
% Predicted	99%	(±5)	101%	(±5)	95%	(±6)
Z‐score	−0.07	(−0.54, 0.44)	0.10	(−0.43, 0.51)	−0.67	(−0.87, −0.07)
Abnormal (*n*, %)	1	(2%)	0	(0%)	1	(11%)
FEF25_75						
% Predicted	98%	(±17)	100%	(±17)	87%	(±17)
Z‐score	−0.18	(−0.61, 0.47)	−0.14	(−0.42, 0.55)	−0.49	(−1.02, −0.12)
Abnormal (*n*, %)	6	(13%)	4	(10%)	2	(22%)
**Body plethysmography**	*n* = 39		*n* = 32		*n* = 7	
TLC						
% Predicted	94%	(±12)	93%	(±11)	100%	(±13)
Z‐score	−0.69	(−1.23, 0.47)	−0.86	(−1.28, 0.17)	−0.05	(−0.94, 0.83)
Abnormal (*n*, %)	5	(13%)	5	(16%)	0	(0%)
RV						
% Predicted	93%	(±42)	87%	(±30)	124%	(±74)
Z‐score	−0.20	(−0.80, 0.43)	−0.26	(−0.80, 0.41)	0.42	(−0.93, 1.83)
Abnormal (*n*, %)	7	(18%)	6	(19%)	1	(14%)
RV/TLC						
Z‐score	−0.18	(−0.87, 0.55)	−0.20	(−0.85, 0.47)	0.44	(−1.04, 2.05)
Abnormal (*n*, %)	3	(8%)	1	(3%)	2	(29%)
sGaw	1.42	(0.97, 1.76)	1.47	(0.98, 1.82)	1.16	(0.83, 1.42)
**Cardiopulmonary Exercise Test**	*n* = 40		*n* = 32		*n* = 8	
*Cardiovascular responses*						
Peak HR (bpm)	185	(175, 192)	185	(175, 192)	177	(161, 198)
Z‐score peak HR	−0.78	(–1.89, 0.09)	−0.75	(−1.54, −0.03)	−1.28	(−4.41, 1.02)
Heart rate reserve (HRR) (bpm)	26	(16, 34)	26	(16, 34)	28	(7, 46)
Abnormal (low) HRR (*n*, %)	10	(25%)	7	(22%)	3	(38%)
Peak oxygen pulse (%)	99	(81, 113)	100	(84, 113)	92	(79, 112)
Abnormal peak O2pulse (*n*, %)	5	(13%)	4	(13%)	1	(13%)
Peak systolic pressure (mmHg)	159	(133, 192)	164	(134, 192)	148	(121, 202)
Abnormal peak Psys (*n*, %)	2	(5%)	2	(6%)	0	(0%)
Peak diastolic pressure (mmHg)	65	(57, 72)	65	(57, 73)	60	(58, 72)
ΔV'O2/ΔWR (mL/min/Watt)	9.8	(9.1, 11.3)	9.8	(9.2, 11.1)	9.6	(8.9, 13.2)
Z‐score ΔV'O2/ΔWR	0.57	(−0.04, 1.40)	0.89	(0.27, 1.49)	−0.23	(−1.46, 0.30)
Abnormal ΔV'O2/ΔWR (*n*, %)	2	(5%)	1	(3%)	1	(13%)
*Respiratory responses*						
Breathing reserve (%)	41	(31, 52)	39	(30, 55)	47	(34, 50)
Z‐score breathing reserve	1.07	(0.48, 1.72)	0.93	(0.43, 1.99)	1.25	(0.67, 1.53)
Abnormal BR (*n*, %)	6	(15%)	5	(16%)	1	(13%)
VT/VC (%)	45	(42, 50)	45	(42, 50)	44	(35, 50)
Abnormal (high) VT/VC (*n*, %)	0	(0%)	0	(0%)	0	(0%)
Flow limitation (TiTtot ratio, %)	48	(46, 51)	49	(47, 51)	47	(46, 49)
Z‐score TiTot ratio	−0.26	(−0.85, 0.61)	−0.02	(−0.74, 0.86)	−0.64	(‐1.59, −0.10)
Abnormal TiTot ratio (*n*, %)	3	(8%)	2	(6%)	1	(13%)
*Breathing regulation (from CNS)*						
Peak breath frequency (b/min)	47	(40, 52)	47	(41, 52)	46	(38, 51)
Z‐score BF	−0.22	(−0.97, 0.37)	−0.29	(−1.11, 0.29)	−0.09	(−0.75, 1.31)
Abnormal BF (*n*, %)	3	(8%)	2	(7%)	1	(13%)
Abnormal BF slope	6	(15%)	4	(13%)	2	(25%)
Abnormal PETCO2 slope	5	(13%)	3	(9%)	2	(25%)
*Deconditioning*						
V'O2‐AT (%)	47	(39, 59)	47	(41, 59)	44	(33, 80)
Abnormal V'O2‐AT (*n*, %)	11	(28%)	8	(25%)	3	(38%)
V'O2 max (%)	92	(67, 101)	96	(69, 103)	85	(64, 98)
Abnormal V'O2 max (*n*, %)	18	(45%)	14	(44%)	4	(50%)
*Gas exchange and ventilation‐perfusion matching*						
Peak SpO2 (%)	97.9	(96.4, 98.7)	97.8	(96.4, 98.1)	98.7	(96.6, 99.0)
Abnormal SpO2 (*n*, %)	1	(3%)	0	(0%)	1	(13%)
VE/VCO2‐AT	27.9	(26.4, 30.3)	27.5	(26.4, 30.0)	31.4	(26.1, 32.3)
Z‐score VE/VCO2‐AT	0.43	(−0.47, 1.35)	0.23	(−0.53, 1.16)	1.68	(1.21, 2.18)
Abnormal VE/VCO2‐AT (*n*, %)	3	(8%)	1	(3%)	2	(25%)
Peak RER	1.10	(1.03, 1.15)	1.09	(1.03, 1.15)	1.11	(1.06, 1.23)
Z‐score peak RER	−0.86	(−1.59, 0.06)	−0.87	(−1.68, 0.06)	−0.71	(−1.56, 0.66)
Abnormal peak RER (*n*, %)	8	(20%)	6	(19%)	2	(25%)
PETCO2‐AT	5.0	(4.7, 5.4)	5.1	(4.8, 5.4)	4.6	(4.1, 5.2)
Z‐score PETCO2‐AT	−0.58	(−1.06, 0.41)	−0.20	(−0.96, 0.81)	−2.33	(−2.53, −0.78)
Abnormal PETCO2‐AT (*n*, %)	7	(18%)	2	(6%)	5	(63%)
P(ET‐E)CO2/PETCO2 (%)	29	(26, 31)	30	(25, 32)	27	(26, 28)
Abnormal P(ET‐E)CO2/PETCO2 (*n*, %)	2	(5%)	2	(6%)	0	(0%)

a Data are presented as mean + standard deviation (SD) for %predicted, and median + interquartile range (IQR) for spirometry z‐scores and values from CPET.

Abbreviations: FEV1: forced expiratory volume in 1 s; FVC: forced vital capacity; FEF25_75: forced expiratory flow at 25%–75% of FVC; TLC: total lung capacity; RV: residual volume; sGaw: specific airway conductance. Peak HR: peak heart rate in beats per minute; peak O2pulse: percentage of oxygen transported per heartbeat; ΔV'O2/ΔWR: Delta V'O2/Delta Workrate; relation between the oxygen uptake (V'O2) and increase in work rate (WR); breathing reserve: the difference between the maximal voluntary ventilation (MVV) and the maximum exercise ventilation (VE); VT/VC: tidal volume at maximum exercise/vital capacity; Ti/Ttot = the ratio of inspiratory time to total respiratory cycle time; BF: breathing frequency; PETCO2: End‐tidal CO2 partial pressure; VO2‐AT: oxygen consumption at anaerobic threshold; VO2 max: maximal oxygen consumption; SpO2: blood level saturation; VE/VCO2‐AT: the ratio of minute ventilation to carbon dioxide production; RER: respiratory exchange rate (gas exchange ratio); P(ET‐E)CO2/PETCO2 **=** mixed‐expired and end‐tidal CO2; high value can point to ventilation and perfusion defects (dead space ventilation).

For the MIS‐C group, prebronchodilator spirometry results revealed an obstructive pattern (FEV1/FVC z‐score < −1.645) in 2 (5%) children, which was no longer present postbronchodilator. Plethysmography showed restriction (TLC z‐score < −1.645) in five (16%) children from the MIS‐C group, of whom 2 (5%) also had a low FVC z‐score (< −1.645). Out of the 6 MIS‐C children with lung function test abnormalities, 2 (33%) had pulmonary comorbidities.

For the COVID‐19 group, prebronchodilator spirometry results revealed an obstructive pattern in 2 (22%) children, which was still present in one (11%) child postbronchodilator. Plethysmography revealed a RV/TLC‐ratio above the 95th percentile in two (29%) children from the COVID‐19 group, suggestive of hyperinflation. Out of the 4 COVID‐19 children with lung function test abnormalities, 2 (50%) had pulmonary comorbidities.

### 3.5 Cardiopulmonary Exercise Test

In total, 40 children (56%) performed CPET (32/43 children (74%) MIS‐C group; 8/29 children (28%) COVID‐19 group) (Table 3).

Table 4 shows CPET results per systemic domain. Out of 40 CPETs performed, 3 (7%) were considered suboptimal, indicating that children did not perform at maximal effort. Because this may be due to underlying pathophysiology (e.g. deconditioning), these results were still considered for analyses. A pattern suggestive for deconditioning was the most reported abnormality (30%).

**Table 4:** Cardiopulmonary Exercise Testing Subanalysis of Systemic (Dys)function Patterns.

	Total	MIS‐C	COVID‐19
	*n* = 40	*n* = 32	*n* = 8
**CPET quality of delivered effort**			
Optimal (at maximum capacity)	37 (93%)	31 (97%)	6 (75%)
Suboptimal	3 (7%)	1 (3%)	2 (25%)
**CPET result**			
Normal	21 (53%)	19 (59%)	2 (25%)
Pathologic	19 (47%)	13 (41%)	6 (75%)
**Cardiovascular responses**			
Normal	36 (90%)	28 (88%)	8 (100%)
Pathologic	4 (10%)	4 (12%)	0 (0%)
**Respiratory responses**			
Normal	37 (93%)	30 (94%)	7 (88%)
Pathologic	3 (7%)	2 (6%)	1 (12%)
**Breathing regulation**			
Normal	33 (83%)	28 (88%)	5 (63%)
Pathologic	7 (17%)	4 (12%)	3 (37%)
**Deconditioning**			
Normal	28 (70%)	23 (72%)	5 (63%)
Pathologic	12 (30%)	9 (28%)	3 (37%)
**Gas exchange**			
Normal	34 (85%)	30 (94%)	4 (50%)
Pathologic	6 (15%)	2 (6%)	4 (50%)

*Note:* Data are presented as *n* (%).

Abbreviations: CPET, cardiopulmonary exercise testing; MIS‐C, multisystem inflammatory syndrome.

From the MIS‐C group, 41% children had abnormal CPET results, based on either an abnormal cardiovascular response (12%), abnormal respiratory responses (6%), abnormal breathing regulation (12%), signs of deconditioning (28%), and/or abnormal gas exchange (6%). Thirteen percent of children reported abnormalities in multiple domains. Out of the 13 MIS‐C children who had CPET abnormalities, 1 (8%) had pulmonary comorbidities.

From the COVID‐19 group, 75% had abnormal CPET results, based on either abnormal respiratory responses (12%), abnormal breathing regulation (37%), signs of deconditioning (37%), and/or abnormal gas exchange (50%). Thirty‐seven percent of children reported abnormalities in multiple domains. Out of the 6 COVID‐19 children who had CPET abnormalities, 3 (50%) had pulmonary comorbidities.

### 3.6 Abnormalities in Clinical Observations, Lung Function Tests, and Cpet

**Figure 2:**
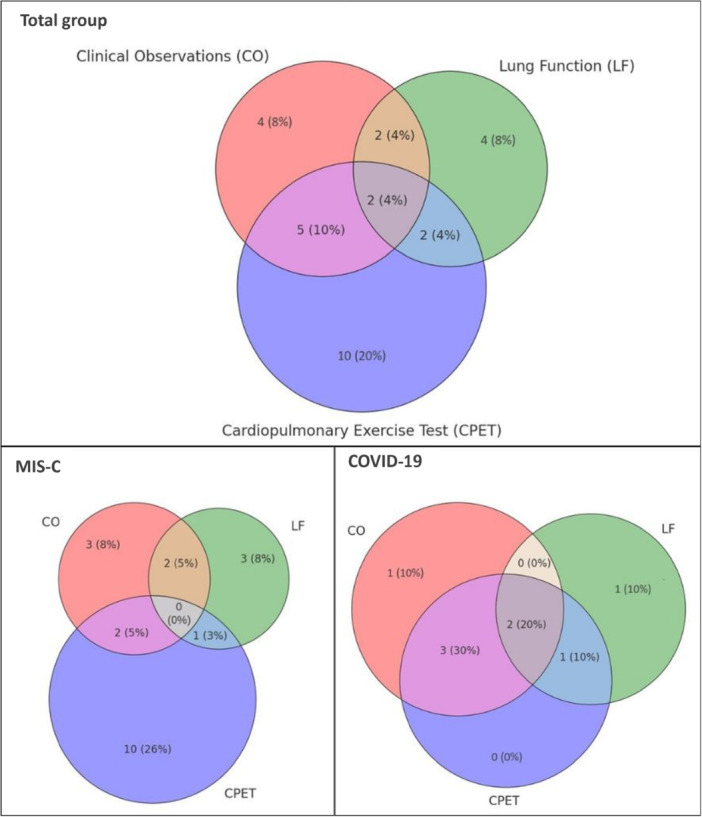
The number of patients with abnormalities in one or more domains at follow‐up visit. CO: clinical observations, the combination of long term respiratory symptoms and/or abnormal physical exam at follow‐up; LF: lung function tests, includes spirometry and body plethysmography; CPET: cardiopulmonary exercise test. Percentages are based on the total number of children who performed LF and/or CPET (*n* = 49 for total group, *n* = 39 for MIS‐C, *n* = 10 for COVID‐19). [Color figure can be viewed at wileyonlinelibrary.com]

Figure 2 and Table S3 displays the number of participants in which abnormalities were found in clinical observations (CO), which is the combination of long‐term respiratory symptoms and/or abnormal physical exam at follow‐up, in lung function tests (LF), and in CPET. For the total group, 29/49 (59%) children showed abnormalities in one or more domain. For the MIS‐C group, 16/39 (41%) children showed abnormalities in one domain, of which CPET was most common (26%), and 5 children (13%) showed abnormalities in two domains. Most children with MIS‐C who showed CPET abnormalities, did not report any respiratory long‐term symptoms or LF abnormalities (10/13; 77%).

For the COVID‐19 group, 2/10 (20%) children had abnormalities in one domain, 4 (40%) in two domains, and 2 (20%) in all three domains. Children with COVID‐19 who showed CPET abnormalities, all reported either long‐term symptoms, LF abnormalities, or both.

### 3.7 Pulmonary Comorbidities in the COVID‐19 Group

Of the COVID‐19 group, 45% of children reported pulmonary comorbidities (Table 1). To evaluate the influence of previous pulmonary disease on long‐term symptoms, spirometry, and CPET, descriptive analyses were performed for two groups: children with COVID‐19 without pulmonary comorbidities, and children with COVID‐19 with pulmonary comorbidities. Results can be found in Table S4, and show a trend towards higher symptom burden and more abnormal functional tests among children without previous pulmonary disease. However, statistical analyses were not performed due to small sample sizes.

## 4 Discussion

In this prospective study, one in five children with a history of MIS‐C or severe COVID‐19, suffered from respiratory symptoms 8 months after hospital presentation. HRQoL was impaired in almost a quarter of participants compared to scores in healthy Dutch peers. Long‐term respiratory symptoms and a lower HRQoL were more frequent in children presenting with COVID‐19, compared to MIS‐C, even after correction for respiratory comorbidities. In both groups, fatigue was the most commonly reported long‐term symptom, with a higher incidence in the MIS‐C group. For children from the MIS‐C group, it was more common to have abnormal functional tests without any long‐term respiratory symptoms. Children from the COVID‐19 group, who were old enough to perform functional tests, often had long‐term respiratory symptoms in combination with abnormal LF and/or CPET.

This study is one of the first prospective studies describing follow‐up of both MIS‐C and severe COVID‐19 patients. By combining clinical data, spirometry results, and CPET results, we give an insight into shared and distinct long‐term outcomes of MIS‐C and COVID‐19.

In MIS‐C, pulmonary sequelae are less commonly reported than cardiovascular sequelae. The percentage of patients with persistent respiratory complaints after MIS‐C in our study is comparable with other studies [30, 31]. Few other studies reported spirometry results during follow‐up of MIS‐C patients, and our study includes the largest series of spirometry results published so far. In one series of 14 children with MIS‐C, FVC values were slightly reduced compared to controls, while other values were normal [32]. In our study, FVC was abnormal in 5%, and restriction was found in 16% of patients using body plethysmography. Two other series found no abnormalities in spirometry during follow‐up [33, 34]. CPET results of the current study cohort were comparable to other MIS‐C cohorts, showing a deconditioning pattern as the most important abnormality, found in 30%–100% of cases [32, 35, 36, 37]. Abnormalities in cardiovascular responses were found in 12% of children from our MIS‐C group. In most MIS‐C guidelines, exercise is not recommended in the first months after hospital admission, an advice based on guidelines concerning cardiomyopathy. This may explain some of the deconditioning patterns found in our MIS‐C group, which is often found after long periods of physical inactivity. Timing of safe return to exercise after MIS‐C therefore deserves more attention and scientific substantiation. Some authors have hypothesized that CPET may be helpful in this decision [38].

In the COVID‐19 group, 41% children reported long‐term respiratory complaints such as (exertional) dyspnea and cough. These findings are in line with the most commonly described pulmonary sequelae of acute SARS‐CoV‐2 infection in children found in other studies [15, 39]. Long‐term fatigue was present in a quarter of children with severe COVID‐19, however, chronic fatigue is a difficult symptom to objectify in young children, and may therefore be underreported in this group. The COVID‐19 group largely consisted of younger children, who showed a trend towards a higher incidence of long‐term respiratory complaints. In older children, we found that most patients with long‐term symptoms also had abnormal lung function tests, and only few had abnormal spirometry results without any complaints.

Based on spirometry (FEV1, FEV1/FVC, and FEF25‐75) values, there may be a cautious trend towards peripheral airway obstruction in the severe COVID‐19 group. In a recent systematic review investigating lung function abnormalities after pediatric SARS‐CoV‐2 infection, the meta‐mean of FEV1, FVC, and FEV25‐75 was normal, with no significant reversibility [18]. Only two out of the eight articles included children with follow‐up after hospital admission for SARS‐CoV‐2 infection: Ipek et al. [40] showed lower FEV1 and FEV1/FVC percentages in the patient group, but did not describe reported complaints; while Boguslawski et al. [41] reported that 17% of patients suffered from persistent respiratory complaints, with normal spirometry results in all. Our results and those from previously mentioned studies, imply that standard lung function testing in the clinical follow‐up of patients with COVID‐19 does not seem necessary in patients without complaints. However, children below 4 years old did not perform lung function tests, despite them reporting most long‐term respiratory symptoms. It is likely their initial disease presentation was related to viral bronchiolitis due to SARS‐CoV‐2, and we know that lung function trajectories can be impacted by bronchiolitis caused by other viral pathogens [10]. Therefore, longer follow‐up studies are needed to assess lung function trajectories in children who suffered from severe COVID‐19 at a young age.

Despite the small number of children in the COVID‐19 group who performed CPET, there were abnormalities in 75%, due to abnormal gas exchange, breathing regulation (e.g. hyperventilation), and/or deconditioning patterns, which are difficult to assess with only spirometry and body plethysmography. CPET may therefore have additional value in objectively monitoring recovery after severe COVID‐19 [42].

Children in our study who reported persistent complaints at follow‐up, fulfill the WHO definition for Pediatric Post‐COVID‐19 Condition (PPCC). A recent study showed that for children with PPCC after non‐severe acute SARS‐CoV‐2 infection [43], CPET results were abnormal in 90.2%, which is also frequently demonstrated in adults [44]. The authors hypothesized that certain abnormalities found during CPET, may reflect pathophysiological mechanisms for PPCC, such as dysautonomia (abnormal breathing regulation and heart rate variability [45]), muscle deconditioning, and pulmonary vasculopathy (abnormal VE/VCO2 and PETCO2 at anaerobic threshold). Some of these findings overlap with abnormalities found in our cohort, which may be an indication of similar dysfunctional physiological processes after SARS‐CoV‐2 infection. This is however speculative, and larger studies are needed which compare CPET in children after non‐severe and severe COVID‐19 and MIS‐C. However, caution is warranted, since CPET itself may also have a negative impact on children with a PPCC‐phenotype including post‐exertional malaise (PEM), in which performing excessive exercise may worsen PEM symptoms [46]. For our cohort, at the time of data collection, PEM was not yet recognized as post‐COVID‐19 symptom, and we therefore unfortunately did not include it in our symptom questionnaire.

Strengths of this study are the inclusion of children of all ages with MIS‐C and severe COVID‐19 infection, its prospective study method, the combination of clinical data collection and extensive lung function testing, and the number of MIS‐C patients included.

This study has some limitations. The number of patients who performed lung function testing was low in the COVID‐19 group, due to young age. Furthermore, since this was a real‐life study, we have no pre‐COVID‐19 data of these participants to compare our results with, meaning we cannot be certain that some of the abnormalities found during lung function testing were not pre‐existent. Additionally, we do not know what effect the pandemic restrictions may have had on long‐term symptoms [47]. Furthermore, given that 62% of our COVID‐19 group reported comorbidities, our findings should not be generalized to the broader pediatric COVID‐19 population, but instead inform clinical follow‐up in children with severe disease warranting hospitalization. Last, only 57% children from the COPP‐study chose to participate in the follow‐up study. Therefore, it may have occurred that mainly participants with remaining symptoms were included, missing out on “healthier” children, or in contrast, children who were still too ill might have denied to participate because of their symptoms. Both may have caused a selection bias, which lowers the overall generalizability.

In conclusion, long‐term respiratory sequelae can occur after both MIS‐C and severe COVID‐19. Long‐term respiratory symptoms and impaired HRQoL were more frequent in the severe COVID‐19 group, whereas fatigue was the most common long‐term symptom in both groups, with a higher incidence in MIS‐C. Abnormalities in functional tests were prevalent in children with COVID‐19, often corresponding with long‐term symptoms. In contrast, for the MIS‐C group, CPET often showed abnormalities without the presence of lung function alterations or respiratory complaints. Longer follow‐up studies need to focus on long‐term sequelae and lung function trajectories of severe COVID‐19 in young infants and toddlers. Clinicians should be aware of the relationship between respiratory complaints and a history of severe COVID‐19 or MIS‐C, and should instruct patients to seek additional care when symptoms do not normalize after hospital admission.

## Author Contributions

**Lieke C. E. Noij:** methodology, data curation, investigation, validation, formal analysis, visualization, writing – original draft, writing – review and editing. **Caroline L. H. Brackel:** conceptualization, project administration, funding acquisition, writing – review and editing, writing – original draft; methodology. **Mariëlle W. Pijnenburg:** conceptualization, writing – review and editing, investigation, methodology, data curation. **Michiel A. G. E. Bannier:** conceptualization, methodology, investigation, data curation, writing – review and editing. **Sanne F. Kloosterman:** methodology, investigation, writing – review and editing. **Joost G. van den Aardweg:** conceptualization, writing – review and editing. **Arjen Pelgröm:** writing – review and editing. **Irene Kuipers:** conceptualization, writing – review and editing. **Erik G. J. von Asmuth:** data curation, methodology, writing – review and editing. **Emilie P. Buddingh:** conceptualization, methodology, funding acquisition, writing – review and editing. **Miriam G Mooij:** conceptualization, writing – review and editing. **Angelique M. A. M. Winkel:** investigation, writing – review and editing. **Lotte Haverman:** software, resources, writing – review and editing. **Lorynn Teela:** resources, writing – review and editing. **Anke H. Maitland van der Zee:** writing – review and editing, supervision. **Johannes B. Goudoever:** supervision, writing – review and editing. **Simone Hashimoto:** conceptualization, methodology, data curation, investigation, supervision, project administration, writing – review and editing. **Suzanne W. J. Terheggen‐Lagro:** conceptualization, methodology, supervision, funding acquisition, project administration, resources, writing – review and editing.

## Ethics Statement

The study was evaluated and approved by the local medical regional ethics committee (METC Amsterdam, reference number N20.043 NL7473696.018.20). Written informed consent was obtained before or at the start of the study visit from caregivers of children < 12 years old and all children older than 12 years and/their caregivers.

## Conflicts of Interest

LCEN, MWP, MAGEB, SFK, JGvA, AP, IK, EGJvA, MGM, AMAMW, LH, LT, SH, and SWJT‐L have nothing to declare. CLHB received research grants from Stichting Astmabestrijding and Stichting Prinses Irene Christina Brader stichting. EPB received research grants from the #wakeuptocorona crowdfund initiative of the Bontius Stichting and Leiden University Fund, and from ZonMw (10430072110007 and 10430102110009). AHM‐Z is the PI of a public private consortium [P4O2 (Precision Medicine for More Oxygen)] sponsored by Health Holland involving many private partners that contribute in cash and/or in kind (AbbVie. Boehringer Ingelheim, Breathomix, Clear, Fluidda, Ortec Logiqcare, Olive, Philips, Quantib‐U, Smartfish, Clear, SODAQ, Thirona, Roche, TopMD, Novartis, RespiQ). She received unrestricted research grants from GSK and Boehringer Ingelheim and received the Vertex innovation award grant, and had honoraria paid to institution by GSK, Boehringer Ingelheim and Astra Zeneca. AHM‐Z is also the chair of DSMB of a study on BPD in neonates and the president of FIGON (Federation Innovative drug research in the Netherlands). JBvG has received a grant from Danone Research and has a patent planned for amino acid composition of infant formulas. He is a member of the national health council (unpaid) and the director of the national Human Milk Bank (unpaid).

## Supporting information

PPUL‐24‐1295 supplements R1 clean.

## Data Availability

The data that support the findings of this study are available from the corresponding author upon reasonable request.
