# Supplementary material for: Pulmonary Sequelae of Severe Acute COVID‐19 and Multisystem Inflammatory Syndrome (MIS‐C) in Dutch Children
Source: Pediatr Pulmonol. 2025 Dec 15;60(12):e71426. doi: 10.1002/ppul.71426 (PMC12706180; doi:10.1002/ppul.71426)
Supplement: Supplementary file 1 — PPUL‐24‐1295 supplements R1 clean. [file PPUL-60-0-s001.docx]

**Supplementary materials**

**Appendix 1: pulmonary function testing and measurement of health related quality of life.**

*Symptom questionnaires*

If children or parents indicated that a symptom was present at follow-up, the following questions were asked:

| **Question** | **Answer which was considered “positive” for presence of long-term symptom** |
| --- | --- |
| Has your child experienced [symptom] in the past two weeks? | Yes |
| How would you qualify [symptom]? | Very severe, Severe, Quite severe |
| Did your child already experience [symptom] before hospitalization for MIS-C or COVID-19? | No |
| Has [symptom] disappeared for long stretches of time between discharge from the hospital and now? | No |

Symptoms were only considered “positive” when all answers to the questions were “positive”.
 *Health Related Quality of Life measures*

Health-related quality of life (HRQOL) was assessed by the Dutch Pediatric Quality of Life Inventory 4.0 (PedsQL 4.0), and was self-reported by children aged 8-18 years old. The PedsQL 4.0 encompasses four domains of HRQoL: Physical Health (8 items), Emotional Functioning (5 items), Social Functioning (5 items), and School Functioning (5 items)^1^. Employing a recall period of one week, respondents rated items on a scale from 1 (“Never a problem”) to 5 (“Almost always a problem”). These responses were then linearly transformed to a 0–100 scale, with higher scores indicating better functioning. The Total PedsQL score was calculated as the mean of all items across the entire questionnaire, also ranging from 0 to 100. The Psychosocial Functioning PedsQL score was calculated as the mean of the Emotional, Social and School scores. Validation studies have confirmed the suitability of the Dutch PedsQL for clinical use in the Netherlands^2^. Cut-off points for impaired HRQoL by the PedsQL 4.0 were set at 1 standard deviation (SD) below the population mean^1^.

*Assessment of spirometry*

Predicted normal values were calculated according to Global Lung Initiative standards.^3^ Measurements of interest were the percentages predicted (%pred) and Z-scores of:

| FEV1 | Forced expiratory volume in 1 second |
| --- | --- |
| FVC | Forced vital capacity |
| FEV1/FVC | Forced expiratory volume in 1 second / Forced vital capacity , also known as Forced Expiratory Ratio (FER) |
| FEF25_75 | Forced expiratory flow at 25–75% of FVC |
| FEV1% reversibility | Reversibility of predicted FEV1 before and after 400 mcg of albuterol |
| FVC% reversibility | Reversibility of predicted FVC before and after 400 mcg of albuterol |

In children older than 8 years, plethysmography was conducted in addition to spirometry. Measurements of interest were the absolute value, percentages predicted and/or Z-scores of:

| TLC | Total lung capacity |
| --- | --- |
| RV | Residual volume |
| RV/TLC | Residual Volume / Total lung capacity ratio |
| SGaw | Specific airway conductance |

Interpretation of spirometry and body plethysmography results according to the Global Lung Initiative Standards.^3^

| Restriction | TLC z-score ≤ -1.65, and/or; FVC z-score ≤ -1.65 |
| --- | --- |
| Obstruction | FEV1/FVC z-score ≤ -1.65 |
| Mixed ventilatory impairment | FEV1/FVC z-score ≤ -1.65 and TLC z-score ≤ -1.65 |
| Bronchodilator response | ≥10% of predicted value in FEV1 |
| Hyperinflation | RV/TLC z-score ≥ 1.65 |

*Assessment of CPET*

Standardized CPET by cycle ergometry was performed using the Cooper protocol, in children aged 6 years or older.^4^ Measurements were performed at rest, during progressive exercise up to maximal exertion, and during recovery. First, the test was judged on the quality of delivered effort. The quality of the delivered effort was considered sufficient if at least one out of three requirements below was met:

| **Abbreviation** | **Outcome** | **Unit** | **Maximum effort if:** | **Measures:** |
| --- | --- | --- | --- | --- |
| RER | Respiratory exchange ratio |  | RER >1.14 | The gas exchange ratio |
| HRR | Heart rate reserve | min^-1^ | HRR <15 min^-1^ | Maximal predicted heart rate minus maximal reached heart rate |
| BR | Breath reserve | % | BR <25% | The difference between the maximal voluntary ventilation (MVV=FEV1x40)) and the maximum exercise ventilation (VE) |

In addition, the following CPET measurements were studied. All z-scores were calculated based on age and sex, using a reference group of healthy Dutch children^5^:

| **Abbreviation** | **Outcome** | **Unit** | **Abnormal if:** | **Measures:** |
| --- | --- | --- | --- | --- |
| HR | Peak heart rate | Beats per minute (BPM) | Z-score ≥1.96 |  |
| O2pulse | Peak oxygen pulse | % predicted | <80% | Percentage of oxygen transported per heartbeat |
| ΔV’O2/ΔWR | Ratio of delta V’O2 to delta work rate | mL/min/Watt | <8.3 | Relation between the oxygen uptake (V’O2) and increase in work rate |
| VT/VC | Ratio of tidal volume at maximum exercise to vital capacity | % | <45% or >65% |  |
| Ti/Tot | Ratio of inspiratory time to total respiratory cycle time | % | <45% | Expiratory flow limitation |
| BF * | Peak breath frequency | Breaths per minute | Z-score ≥1.96 |  |
| V’O2-AT | Oxygen uptake at anaerobic threshold | % predicted | <40% |  |
| V’O2-max | Oxygen uptake at maxim effort | % predicted | <85% |  |
| SpO2 | Peak arterial blood oxygen saturation | % | <95% |  |
| Psys | Peak systolic blood pressure | mmHg | Z-score ≥1.96 |  |
| Pdias | Peak diastolic pressure | mmHg |  |  |
| VE/VCO2 at AT | Ventilation minute for carbon dioxide production at anaerobic threshold | kPa | Z-score ≥1.96 | Ventilation efficiency |
| PETCO2-AT | End-tidal partial pressure for carbon dioxide | kPa | Z-score ≥1.96 |  |
| P(ET-E)CO2/ PETCO2 | Ratio of mixed-expired and end-tidal CO_2_ | % | >35% | Relative alveolar dead space ventilation. |

* In the Cycleplotter graph, BF and PETCO2 were examined by three independent researchers and classified as normal or abnormal slope.

Next, the CPET results were classified as normal or abnormal per function pattern. The following decisions rules were applied:

| Overall CPET result |  |
| --- | --- |
| Normal, if: | No abnormalities in any domain. |
| Cardiovascular responses |  |
| Abnormal if: | Abnormal O_2_ pulse, delta Psys, and/or ΔVO2/ΔWR |
| Respiratory responses |  |
| Abnormal if: | High VT/VC and/or abnormal Ti/Tot max |
| Breathing regulation (central nervous system) |  |
| Abnormal if: | High BF, and/or abnormal BF- and/or PETCO_2_ -slope |
| Deconditioning |  |
| Abnormal if: | Abnormal VO2-AT |
| Gas exchange and ventilation-perfusion matching |  |
| Abnormal if: | High RER, abnormal delta SpO2, VE/VCO_2_ at AT, PETCO_2_ at AT, and/or P(ET-E)CO_2_/ PETCO_2_ |

**Appendix 2: supplementary tables**

**Table S1. Long-term respiratory sequelae in children, according to age group**

|  | Total | Children aged  0-5 years | Children aged  6-18 years | p-value* |
| --- | --- | --- | --- | --- |
|  | N=72 | N=21 | N=51 |  |
| Acute clinical presentation |  |  |  | **<0.01** |
| MIS-C | 43 (60%) | 4 (19%) | 39 (77%) |  |
| COVID-19 | 29 (40%) | 17 (81%) | 12 (23%) |  |
| Long term complaints:  Respiratory symptoms | 14 (19%) | 7 (33%) | 7 (14%) | 0.07 |
| Dyspnea during exercise | 11 (15%) | 4 (19%) | 6 (12%) | 0.44 |
| Dyspnea while resting | 3 (4%) | 1 (5%) | 2 (4%) | 0.90 |
| Cough | 7 (10%) | 6 (29%) | 0 (0%) | NA |
| Wheezing | 2 (3%) | 1 (5%) | 0 (0%) | NA |
| Long term complaints:  Fatigue | 26 (36%) | 4 (19%) | 21 (41%) | 0.08 |
| Long term complaints:  Includes all symptoms^+^ | 56 (78%) | 18 (86%) | 32 (63%) | 0.07 |

Data are presented as n (%), unless indicated otherwise. NA: not applicable.
* P-value for ordinal variables from logistic regression (with covariate pulmonary comorbidities), p-value for continuous variables from ANCOVA (with covariate pulmonary comorbidities).

After recognition of PPCC in early 2021, an amendment of the COPP2 study included complaints from more organ systems, of which results are shown in Table S2.

**Table S2. Long term non-respiratory sequelae in children with either COVID-19 or MIS-C**

|  | Total | MIS-C | COVID-19 | p-value* |
| --- | --- | --- | --- | --- |
|  | N=72 | N=43 | N=29 |  |
| Long term complaints:  Includes all symptoms | 50 (69%) | 26 (62%) | 23 (79%) | 0.19 |
| Non-respiratory symptoms | N=51 | N=37 | N=14 |  |
| *Neurological symptoms* | 22 (38%) | 18 (49%) | 3 (21%) | **0.02** |
| Concentration loss | 14 (27%) | 12 (32%) | 2 (14%) |  |
| Headache | 12 (24%) | 9 (25%) | 3 (21%) |  |
| Dizziness | 4 (8%) | 3 (8%) | 1 (7%) |  |
| Difficulties with memory | 4 (8%) | 3 (8%) | 1 (7%) |  |
| Loss of taste | 6 (12%) | 3 (8%) | 3 (21%) |  |
| Loss of smell | 5 (10%) | 2 (6%) | 3 (21%) |  |
| *Gastro-intestinal symptoms* | 19 (37%) | 11 (31%) | 8 (57%) | 0.73 |
| Abdominal pain | 13 (25%) | 7 (19%) | 6 (43%) |  |
| Loss of appetite | 10 (20%) | 5 (14%) | 5 (36%) |  |
| Diarrhea | 7 (14%) | 2 (6%) | 5 (36%) |  |
| *Systemic symptoms* | 14 (27%) | 10 (28%) | 4 (29%) | 0.35 |
| Abnormal sweating | 8 (16%) | 5 (14%) | 3 (21%) |  |
| Recurrent fever | 4 (8%) | 2 (6%) | 2 (14%) |  |
| Skin lesions | 4 (8%) | 2 (6%) | 2 (14%) |  |
| Myalgia | 2 (4%) | 2 (6%) | 0 (0%) |  |

Data are presented as n (%), unless indicated otherwise. * P-value for ordinal variables from logistic regression (with covariate pulmonary comorbidities), p-value for continuous variables from ANCOVA.

**Table S3. Abnormal results per participant that performed functioning tests**

| **Participant** | **MIS-C or COVID-19** | **Clinical observations** | **Lung function testing** | **CPET** | **Number of abnormal results** |
| --- | --- | --- | --- | --- | --- |
| Participant 1 | MIS-C | normal | normal | abnormal | 1 |
| Participant 2 | COVID-19 | normal | normal | normal | 0 |
| Participant 3 | MIS-C | abnormal | normal | abnormal | 2 |
| Participant 4 | MIS-C | normal | normal | normal | 0 |
| Participant 5 | MIS-C | normal | normal | abnormal | 1 |
| Participant 6 | MIS-C | normal | abnormal | normal | 1 |
| Participant 7 | MIS-C | normal | abnormal | abnormal | 2 |
| Participant 8 | MIS-C | abnormal | normal | normal | 1 |
| Participant 9 | MIS-C | abnormal | abnormal | normal | 2 |
| Participant 10 | MIS-C | normal | normal | abnormal | 1 |
| Participant 11 | MIS-C | abnormal | abnormal | N.A | 2 |
| Participant 12 | MIS-C | normal | normal | normal | 0 |
| Participant 13 | COVID-19 | abnormal | normal | N.A | 1 |
| Participant 14 | MIS-C | normal | abnormal | N.A | 1 |
| Participant 15 | MIS-C | normal | normal | normal | 0 |
| Participant 16 | MIS-C | normal | normal | N.A | 0 |
| Participant 17 | MIS-C | normal | normal | normal | 0 |
| Participant 18 | MIS-C | normal | normal | abnormal | 1 |
| Participant 19 | MIS-C | normal | normal | abnormal | 1 |
| Participant 20 | MIS-C | normal | normal | normal | 0 |
| Participant 21 | MIS-C | normal | normal | abnormal | 1 |
| Participant 22 | MIS-C | normal | normal | normal | 0 |
| Participant 23 | MIS-C | normal | normal | N.A | 0 |
| Participant 24 | MIS-C | normal | normal | abnormal | 1 |
| Participant 25 | MIS-C | normal | normal | normal | 0 |
| Participant 26 | MIS-C | normal | normal | abnormal | 1 |
| Participant 27 | MIS-C | normal | normal | normal | 0 |
| Participant 28 | MIS-C | normal | normal | abnormal | 1 |
| Participant 29 | COVID-19 | abnormal | N.A | abnormal | 2 |
| Participant 30 | MIS-C | normal | normal | normal | 0 |
| Participant 31 | MIS-C | normal | normal | normal | 0 |
| Participant 32 | COVID-19 | abnormal | normal | abnormal | 2 |
| Participant 33 | COVID-19 | abnormal | abnormal | abnormal | 3 |
| Participant 34 | COVID-19 | abnormal | abnormal | abnormal | 3 |
| Participant 35 | COVID-19 | normal | abnormal | abnormal | 2 |
| Participant 36 | COVID-19 | normal | abnormal | normal | 1 |
| Participant 37 | MIS-C | abnormal | normal | abnormal | 2 |
| Participant 38 | MIS-C | normal | normal | normal | 0 |
| Participant 39 | MIS-C | abnormal | normal | normal | 0 |
| Participant 40 | MIS-C | normal | normal | abnormal | 1 |
| Participant 41 | MIS-C | abnormal | normal | normal | 1 |
| Participant 42 | COVID-19 | abnormal | normal | abnormal | 2 |
| Participant 43 | MIS-C | normal | normal | N.A | 0 |
| Participant 44 | MIS-C | normal | normal | N.A | 0 |
| Participant 45 | MIS-C | normal | normal | normal | 0 |
| Participant 46 | COVID-19 | normal | normal | N.A | 0 |
| Participant 47 | MIS-C | normal | abnormal | normal | 1 |
| Participant 48 | MIS-C | normal | normal | N.A | 0 |
| Participant 49 | MIS-C | normal | normal | normal | 0 |

Abnormal clinical observations: long term respiratory symptoms and/or abnormal physical exam at follow-up; lung function testing: includes spirometry and plethysmography results; CPET: cardiopulmonary exercise test

**Table S4. COVID-19 group divided into two groups based on pulmonary comorbidities**

|  | Total | Children without pulmonary comorbidities | Children with pulmonary comorbidities |
| --- | --- | --- | --- |
|  | N=29 | N=16 | N=13 |
| Age (median, IQR) | 2 (0, 13) | 1 (0, 14) | 6 (2, 11) |
| Sex |  |  |  |
| Boy | 16 (55%) | 7 (44%) | 9 (69%) |
| Girl | 13 (45%) | 9 (56%) | 4 (31%) |
| ICU admission (yes) | 2 (7%) | 1 (6%) | 1 (8%) |
| Long term complaints:  Respiratory symptoms | 12 (41%) | 8 (50%) | 4 (31%) |
| Dyspnea during exercise | 8 (28%) | 6 (38%) | 2 (15%) |
| Dyspnea while resting | 2 (7%) | 1 (6%) | 1 (8%) |
| Cough | 6 (21%) | 5 (31%) | 1 (8%) |
| Wheezing | 1 (3%) | 0 (0%) | 1 (8%) |
| Long term complaints:  Fatigue | 7 (24%) | 4 (25%) | 3 (21%) |
| Total PedsQL score | *N=10* | *N=5* | *N=5* |
| Abnormal | 6 (60%) | 5 (100%) | 1 (20%) |
| Spirometry | *N=9* | *N=3* | *N=6* |
| Abnormal | 4 (44%) | 2 (67%) | 2 (33%) |
| CPET | *N=8* | *N=4* | *N=4* |
| Abnormal | 6 (75%) | 4 (100%) | 2 (50%) |

*References for supplementary materials*

1. Varni J, Burwinkle T, Seid M, Skarr D. The PedsQL* 4.0 as a Pediatric Population Health Measure: Feasibility, Reliability, and Validity. Ambulatory Pediatrics. 2003;3(6):329-41 DOI: 10.1367/1539-4409(2003)003&lt;0329:TPAAPP&gt;2.0.CO;2.

2. van Muilekom MM, Luijten MAJ, van Oers HA, Conijn T, Maurice-Stam H, van Goudoever JB, et al. Paediatric patients report lower health-related quality of life in daily clinical practice compared to new normative PedsQL(TM) data. Acta Paediatr. 2021;110(7):2267-79 DOI: 10.1111/apa.15872.

3. Stanojevic S, Kaminsky DA, Miller MR, Thompson B, Aliverti A, Barjaktarevic I, et al. ERS/ATS technical standard on interpretive strategies for routine lung function tests. Eur Respir J. 2022;60(1) DOI: 10.1183/13993003.01499-2021.

4. Cooper, CB, Storer TW. Exercise Testing and Interpretation: A Practical Approach. Cambridge University Press. 2009. <https://doi.org/10.1017/CBO9780511545689>.

5. Bongers, B, Hulzebos, E, van Brussel, M, Takken, T. Pediatric Norms for Cardiopulmonary Exercise Testing. Boxpress, Den Bosch. 2021.
